# Ocular blood flow dynamics following sinus rhythm restoration through catheter ablation: laser speckle flowgraphy in patients with persistent atrial fibrillation

**DOI:** 10.1093/ehjimp/qyae071

**Published:** 2024-08-08

**Authors:** Nobuhiko Yamamoto, Makoto Nakano, Kotaro Nochioka, Masayuki Yasuda, Hiroshi Kunikata, Toru Nakazawa, Satoshi Yasuda

**Affiliations:** Departments of Cardiovascular Medicine, Tohoku University Graduate School of Medicine, 1-1, Seiryo-machi, Aoba-ku, Sendai 980-8574, Japan; Departments of Cardiovascular Medicine, Tohoku University Graduate School of Medicine, 1-1, Seiryo-machi, Aoba-ku, Sendai 980-8574, Japan; Departments of Cardiovascular Medicine, Tohoku University Graduate School of Medicine, 1-1, Seiryo-machi, Aoba-ku, Sendai 980-8574, Japan; Department of Ophthalmology, Tohoku University Graduate School of Medicine, Sendai, Japan; Department of Ophthalmology, Tohoku University Graduate School of Medicine, Sendai, Japan; Department of Ophthalmology, Tohoku University Graduate School of Medicine, Sendai, Japan; Departments of Cardiovascular Medicine, Tohoku University Graduate School of Medicine, 1-1, Seiryo-machi, Aoba-ku, Sendai 980-8574, Japan

**Keywords:** LSFG, microcirculation, atrial fibrillation, catheter ablation, ocular blood flow, cognitive impairment

## Abstract

**Aims:**

Laser speckle flowgraphy (LSFG) is a well-established tool renowned for its non-invasive and reproducible assessment of ocular blood flow. While rhythm control therapies, such as catheter ablation (CA), have shown promise in enhancing cognitive function in atrial fibrillation (AF) patients, the acute impact of CA on microcirculatory changes, particularly in ocular blood flow, remains a topic of limited understanding. The present study aims to delve into the potential of LSFG in detecting microcirculatory alterations following the restoration of sinus rhythm (SR) through CA in patients with AF.

**Methods and results:**

We studied 8 paroxysmal AF (Paf) and 20 persistent AF (PeAF) patients (mean age 67 ± 6 years, 26% female) undergoing CA. Ocular blood flow was assessed using LSFG by measuring the mean blur rate (MBR) pre- and post-CA. Post-CA, all PeAF patients achieved SR restoration, resulting in a significant increase in tissue MBR (10.0 ± 2.2 to 10.8 ± 2.9, *P* = 0.021). In contrast, Paf patients showed no significant difference between pre- and post-MBR (12.0 ± 2.7 vs. 11.8 ± 2.6, *P* = 0.76).

**Conclusion:**

LSFG analysis effectively identified microcirculatory changes in patients undergoing CA for PeAF, suggesting that therapeutic interventions targeting the heart may have broader implications for ocular and cerebral health, establishing a novel ‘cardio-oculo-cerebral relationship’.

## Graphical Abstract

**Graphical Abstract:**
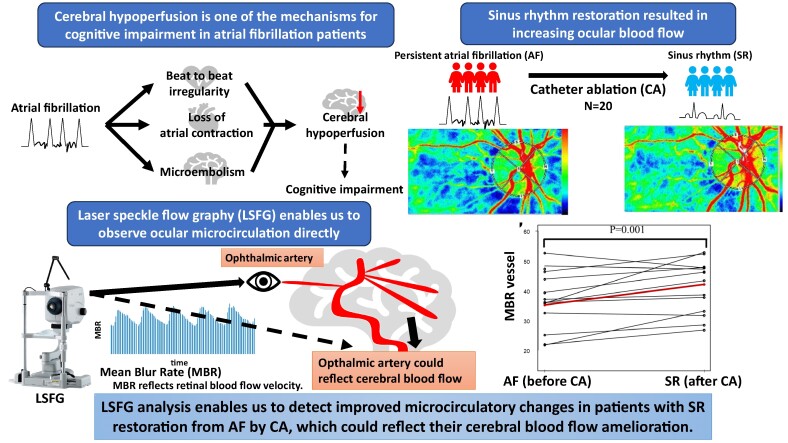

## Introduction

Laser speckle flowgraphy (LSFG) has emerged as an invaluable tool for non-invasive and reproducible analysis of ocular blood flow, contributing significantly to the detection of various ophthalmic diseases.^1^ Notably, LSFG parameters have played pivotal roles in predicting silent cerebral infarction in patients with primary aldosteronism,^2^ highlighting its potential as a predictive marker for systemic cardiovascular disease.

Within the context of atrial fibrillation (AF), rhythm control therapies such as catheter ablation (CA) have demonstrated promise not only in reducing adverse cardiovascular events but also in improving cognitive function.^3–5^ Despite these advancements, the acute impact of CA on microcirculatory changes, particularly in ocular blood flow, remains insufficiently understood. This study therefore aims to explore the capacity of LSFG in detecting microcirculatory alterations resulting from the restoration of sinus rhythm (SR) following CA in patients with AF.

## Methods

The study involved consecutive 28 AF patients [8 paroxysmal AF (Paf) and 20 persistent AF (PeAF)] scheduled for CA and free of structural heart disease, as confirmed by computed tomography, transthoracic echocardiography, and resting electrocardiogram. Before undergoing CA, all patients received anticoagulation therapy with warfarin or a direct oral anticoagulant (DOAC) for a minimum of 1 month, which was continued for at least 3 months post-CA.

CA was conducted under general anaesthesia using propofol and dexmedetomidine. Pulmonary vein isolation (PVI) was performed in all patients using cryo- or radiofrequency (RF) energy, guided by a 3D anatomical mapping system (CARTO3 or Ensite Navx). Electrical cardioversion was administered before energy application in patients with AF rhythm at the beginning of the session. Additional procedures such as posterior wall isolation and non-pulmonary vein trigger ablation, as well as the selection of energy sources, were at the discretion of the operator.

Ocular microcirculation was evaluated using LSFG both before and after CA. The post-ablation assessments were conducted within 2 to 3 days as the acute phase evaluation and ∼6 months later as the chronic phase evaluation. Additionally, pulse waveform parameters derived from mean blur rate (MBR), were analysed, which included blowout score (BOS), blowout time (BOT), and flow acceleration index (FAI). MBR serves as an indicator of retinal blood flow velocity, while BOS quantifies blood flow per heartbeat, BOT measures the percentage of blood flow half-width per heartbeat, and FAI reflects the maximum changes in MBR. We measured MBR in tissue, which specifically represents capillary blood flow, enabling individual comparisons.

Ethical approval for this study was obtained from the Tohoku University Institutional Review Board (2022-1-353), and informed consent was acquired from all participating patients. Statistical analysis involved presenting continuous variables as means ± standard deviation (SD), calculated by Student’s *t*-test for parametric variables and Mann–Whitney *U* test for non-parametric variables. Statistical analyses were conducted using EZR^6^ on R commander version 1.61 (Saitama Medical Centre, Jichi Medical University).

## Results

The characteristics of the 28 consecutive patients, divided into the Paf (*n* = 8) and PeAF (*n* = 20) groups, are shown in *Table 1*. The mean age was 67 ± 6 years, with 7 (26%) being female. Structural heart diseases were present in 9 (45%) of the patients, while 16 (59%) had hypertension, and 7 (26%) had diabetes mellitus. Compared with the Paf group, PeAF patients had significantly lower LVEF (57 ± 11% vs. 68 ± 6%, *P* = 0.014) and tended to have a larger LA volume (75 ± 14 mL vs. 62 ± 21 mL, *P* = 0.08). No specific ocular diseases were identified before or after CA. Following CA, all patients in the PeAF group transitioned from AF to SR, whereas patients in the Paf group maintained SR both before and after CA.

**Table 1:** Characteristics of 28 consecutive patients with persistent atrial fibrillation and paroxysmal atrial fibrillation

	All (*n* = 28)	Persistent (*n* = 20)	Paf (*n* = 8)	*P*-value
Age (years)	67 ± 6	66 ± 6	70 ± 4	0.10
Female sex, *n* (%)	7 (26%)	4 (21%)	3 (37%)	0.63
HT, *n* (%)	16 (59%)	12 (63%)	4 (50%)	0.67
DM, *n* (%)	7 (26%)	3 (31%)	1 (12%)	0.63
CHF, *n* (%)	5 (18%)	5 (26%)	0	0.28
Stroke/TIA, *n* (%)	1 (3%)	1 (6%)	0	1.00
CHADS2-VASc score	2.4 (2–3)	2 (2–3.5)	2 (1.75–2.25)	0.55
β-blocker, *n* (%)	20 (74%)	13 (63%)	7 (87%)	0.63
AAD, *n* (%)	7 (26%)	4 (21%)	3 (0.37)	0.63
ACEi/ARB, *n* (%)	17 (62%)	13 (68%)	4 (50%)	0.41
DOAC, *n* (%)	26 (97%)	18 (94%)	8 (100%)	0.29
Antiplatelet, *n* (%)	0	0	0	1.00
Median days since AF diagnosis (days)	663 ± 917	651 ± 1052	693 ± 526	0.91
LVEF (%)	60 ± 11	57 ± 11	68 ± 6	0.016
LVDd (mm)	47 ± 5	48 ± 5	44 ± 6	0.18
LAD (mm)	40 ± 7	41 ± 6	38 ± 8	0.13
LAVI (mL/m^2^)	41 ± 11	43 ± 11	36 ± 11	0.15
LA volume (4CV) (mL)	71 ± 17	75 ± 14	62 ± 21	0.08
Time from CA to post LSFG (days)	2.2 ± 0.6	2.2 ± 0.6	2.3 ± 0.6	0.78

PeAF, persistent atrial fibrillation; Paf, paroxysmal atrial fibrillation; HT, hypertension; DM, diabetes mellitus; CHF, congestive heart failure; ACEi, angiotensin converting enzyme inhibitor; ARB, angiotensin receptor blocker; AAD, antiarrhythmic drug; DOAC, direct oral anticoagulant; LVEF, left ventricular ejection fraction; LVDd, left ventricular dimension of diastole; LAD, left atrial diameter; LAVI, left atrial volume index; LSFG, laser speckle flowgraphy; CA, catheter ablation.

**Figure 1:**
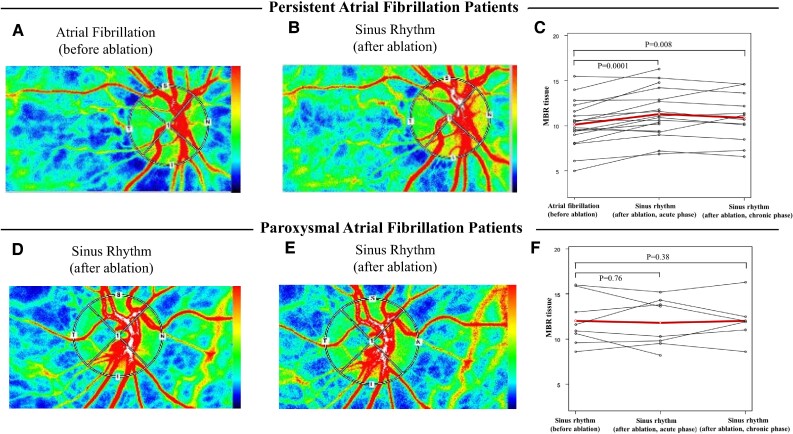
Ocular blood flow changes detected by LSFG analysis in patients undergoing catheter ablation for atrial fibrillation. LSFG uses a laser to assess fundus blood flow, where scattering rays create a distinctive speckled pattern known as the speckle phenomenon. This pattern changes with blood flow, quantified as the MBR. Representative images demonstrate ocular blood flow in patients with AF. (*A–C*) show data from PeAF patients, while (*D–F*) present data from Paf patients. Specifically, (*A* and *D*) show conditions before catheter ablation, and (*B* and *E*) show conditions after catheter ablation in SR. (*C* and *F*) demonstrate changes in MBR. MBR reflects retinal blood flow velocity, with the red-dotted line indicating the average change. Group comparisons were conducted using the Wilcoxon signed-rank test, considering *P* < 0.05 as statistically significant. LSFG, laser speckle flowgraphy; CA, catheter ablation; AF, atrial fibrillation.

LSFG analysis revealed a dynamic enhancement in ocular blood flow upon the restoration of SR in PeAF group patients. Specifically, acute phase analysis showed significant increases in tissue MBR (10.1 ± 2.5 to 11.3 ± 2.5, *P* = 0.0001), indicating marked changes in ocular blood flow after SR restoration (*Figure 1A–C*). Additionally, parameters derived from tissue MBR such as BOS (82.9 ± 4.8 to 76.0 ± 5.0, *P* = 0.0001), BOT (52.5 ± 14 to 48.5 ± 4.2, *P* = 0.04), and FAI (1.00 ± 0.42 to 1.35 ± 0.55, *P* = 0.001) also changed dynamically in PeAF patients following SR restoration after CA. In contrast, as shown in *Figure 1C–E*, Paf patients did not show any statistical difference in pre- and post-tissue MBR during the acute phase analysis (12.0 ± 2.7 vs. 11.8 ± 2.6, *P* = 0.76).

Although only a small number of patients were analysed (*n* = 12), the tendency for improved LSFG was maintained until the chronic phase among PeAF patients (10.0 ± 2.2 to 10.9 ± 2.6, *P* = 0.008). Conversely, there were no differences among Paf patients even in the chronic phase (12.0 ± 2.7 vs. 12.0 ± 2.5, *P* = 0.38).

To elucidate the contributing factors for the improvement of MBR (defined as post-/pre-MBR > 1.0), we performed a multivariate analysis. The following clinical variables were included: age, sex, form of AF, LVEF, LAD, taking angiotensin converting enzyme inhibitor (ACEi)/ angiotensin receptor blocker (ARB), taking β-blocker, and HR change after CA. Using the stepwise AIC method, the form of AF was the only variable that showed a trend towards being a significant predictor for the improvement of MBR, though it did not reach conventional statistical significance (OR 5.5, 95% CI 0.83–34.1, *P* = 0.077).

## Discussion

The novel finding of this study is the capability of LSFG to identify an increase in ocular blood flow, as represented by MBR, among patients undergoing CA for persistent AF. To our knowledge, this is the first study to demonstrate LSFG’s potential to identify acute microcirculatory changes during SR restoration from AF.

Recent studies have suggested that successful CA for AF may contribute to left atrial reverse remodelling and an increase in cerebral blood flow.^3,4,7,8^ This study effectively demonstrates the notable improvement in ocular microcirculation following CA-induced SR restoration, as evidenced by LSFG analysis. The MBR, serving as a direct indicator of blood flow within the ocular fundus, appears particularly responsive to this change.

The retinal vasculature is a unique system of blood vessels in the body, as it can be directly observed from outside the organism. This exceptional characteristic allows for the non-invasive assessment of circulatory dynamics. Recent investigations have highlighted the correlation between ocular blood flow, as detected by LSFG, and regional cerebral oxygen saturation, a valuable parameter related to cerebral oxygen metabolism and cognitive function.^9^ Other studies have reported that reduced cerebral blood flow detected through magnetic resonance imaging (MRI) could pose a dementia risk^5^ and that peak systolic velocity analysed by transcranial Doppler ultrasound might be linked to cognitive function.^10^ Importantly, ocular microcirculation is maintained by the ophthalmic artery, a branch of the carotid artery. Therefore, LSFG-detected ocular blood flow may correspond to that of the cerebral artery and have implications for cognitive function.

While MRI or positron emission tomography are capable of analysing cerebral circulation, they are not certainly suitable for screening or repetitive analysis due to their cost and time-intensive nature. In contrast, LSFG-based ocular blood flow analysis offers a quick, reproducible, and cost-effective approach, making it well-suited for repeated assessments and screening of microcirculation. Collectively, LSFG-based examination of ocular microcirculation holds promise as a valuable screening tool for assessing cerebral circulation and cognitive dysfunction.

Several limitations warrant consideration. First, the study’s small sample size and single-centre approach might limit the generalizability of the findings. Secondly, direct cognitive assessments were not conducted in the present study. Thirdly, the cohort consisted predominantly of relatively young patients, which may affect the applicability of the results to older populations. Lastly, additional investigation is required to determine whether the method of sinus rhythm restoration (electrical cardioversion, ablation, and antiarrhythmic drugs) could influence the outcomes.

## Conclusion

In conclusion, LSFG analysis successfully detected microcirculatory changes in patients undergoing CA for PeAF, indicating the therapeutic effects on the heart might also have implications for the eyes and brain termed as ‘cardio-oculo-cerebral relationship’.

## Data Availability

Data are available on reasonable request.

## Lead author biography

Dr Nobuhiko Yamamoto is a cardiologist working at the Tohoku University Hospital in Sendai, Japan. He is mostly interested in electrophysiology and heart failure, with a special focus on catheter ablation and device therapy.
